# Association Between Parental Anxiety and Depression Level and Psychopathological Symptoms in Offspring With 22q11.2 Deletion Syndrome

**DOI:** 10.3389/fpsyt.2020.00646

**Published:** 2020-07-23

**Authors:** Corrado Sandini, Maude Schneider, Stephan Eliez, Marco Armando

**Affiliations:** ^1^ Developmental Imaging and Psychopathology lab, Faculty of Medicine, University of Geneva, Geneva, Switzerland; ^2^ Clinical Psychology Unit for Intellectual and Developmental Disabilities, Faculty of Psychology and Educational Sciences, University of Geneva, Geneva, Switzerland; ^3^ Center for Contextual Psychiatry, Department of Neurosciences, KU Leuven, Leuven, Belgium

**Keywords:** 22q11.2, parental psychological distress, gene x environment, offspring, anxiety, depression, psychosis

## Abstract

22q11.2 deletion syndrome (22q11DS) is recognized as one of the strongest genetic risk factors for the development of psychopathology, including dramatically increased prevalence of schizophrenia anxiety disorders, mood disorders, and Attention Deficit Hyperactivity Disorder (ADHD). Despite sharing a homogenous genetic deletion, the psychiatric phenotype in 22q11DS still present significant variability across subjects. The origins of such variability remain largely unclear. Levels of parental psychopathology could significantly contribute to phenotypic variability of offspring psychopathology, through mechanisms of gene x gene (GxG) and gene x environment (GxE) interactions. However, this hypothesis has not been explicitly tested to date in 22q11DS. In the present manuscript, we employed a longitudinal design to investigate bi-directional interactions of parental anxiety and depressive symptoms, estimated with Beck Depression Inventory and Beck Anxiety Inventory, and offspring level of psychopathology assessed with a combination of parentally reported Child Behavioral Checklist, Youth Self Report Questionnaire, and Structured Clinical Interviews for Prodromal Syndromes (SIPS). We tested associations in both typically developing healthy controls (HCs) (N = 88 participants; N = 131 time points) and in individuals with 22q11DS (N = 103 participants; N = 198 time points). We observed that 22q11DS individuals with higher levels of parental anxiety and depression presented significant increases in multiple forms of psychopathology, including higher internalizing and externalizing symptoms, as estimated both by parental and self-report questionnaires, along with higher negative and generalized symptoms as measured with the SIPS. Associations for positive and disorganized dimensions of the SIPS were not statistically significant. Purely longitudinal analysis pointed to bi-directional interactions of parental and child psychopathology, with marginally stronger longitudinal associations between early parental anxiety-depression and subsequent child psychopathology. Interestingly, associations between psychopathology across generations were significantly stronger in 22q11DS individuals compared to HCs. Our results show that parental levels of anxiety and depression are associated with levels of offspring psychopathology, particularly in individuals with 22q11DS. These findings point to the existence of GxG or GxE mechanisms, that should be investigated in future work. From a clinical perspective, they highlight a strong rational for the management of parental psychological well-being in 22q11DS.

## Introduction

The 22q11.2 deletion syndrome (22q11DS) is one of the most common recurrent copy number variant disorders occurring in approximately one in 3,000–4,000 live births and up to one in 1,000 pregnancy (1). It is caused by a microdeletion resulting in hemizygosity for approximately 50 genes (2). 22q11DS is associated with a variety of symptoms including physical, social, cognitive, and psychiatric problems (2). In particular, individuals with 22q11DS are characterized by an increased prevalence of neurodevelopmental disorders (25–50%; i.e. attention deficit hyperactivity disorder, autism spectrum disorder, intellectual disability, learning disabilities), anxiety and mood disorders (15–65%), and psychosis spectrum disorders (20–30%) (3, 4). Similar to what has been described in the general population, psychiatric comorbidity is extremely frequent in the context of 22q11DS. For example, it has been shown that individuals with 22q11DS diagnosed with an anxiety disorder are six times more likely to be also diagnosed with a mood and psychosis spectrum disorder compared to those without an anxiety disorder (4). Despite the increased prevalence of psychiatric disorders reported in this population, about 20% of individuals with 22q11DS experience non-clinical levels of psychopathology (5), thus highlighting considerable heterogeneity within the 22q11DS group.

To date, the reasons for this clinical heterogeneity remain unclear and could encompass a complex combination of both gene x gene (GxG) and gene x environment (GxE) interactions. Some studies have recently shown evidence that GxG interactions modulate risk for schizophrenia in 22q11DS (6–8). In particular, Cleynen et al. (6) observed that the presence of common genetic variants outside of the 22q11.2 locus and known to be associated with risk for schizophrenia—also known as the polygenic risk score for schizophrenia—is more frequent in individuals with 22q11DS diagnosed with a psychotic disorder compared to those without psychosis.

With regards to GxE interactions, evidence is more limited but several mice and human studies point toward significant effects (3). For example, a recent study has shown that exposure to stressful life events modulated the risk to experience psychotic symptoms in adolescents and young adults with 22q11DS (9). This study showed also that 22q11DS patients are more sensitive to stressful life events (i.e. which includes parental psychopathology) compared to typically developing offspring, highlight the importance of environmental factors in the pathway to severe mental disorders in 22q11DS. Nevertheless, the study of other important environmental factors, such as specific aspects of the familial environment, has received limited attention. The association between parenting style and behavioral outcome in the offspring diagnosed with 22q11DS has been investigated in two studies (10, 11), both of them showing significant associations. Weisman et al. (12) also investigated dyadic reciprocity during a mother-child interaction and showed significant associations with the level of behavior problems assessed with the Child Behavior Checklist [CBCL (13)].

In the general population, there is substantial evidence that several aspects of the familial environment, such as high family cohesion or a high parental involvement, can act as resilience factors to reduce the risk of psychopathology in vulnerable populations (14). It has also been shown that factors pointing toward lower levels of parental well-being, such as parental stress or the presence of (subclinical) anxiety and depressive symptoms increase the risk of psychopathology in the offspring (15). It should be noted that the vast majority of research focused on parental anxiety and depressive symptoms, which are the most frequent symptoms in the adult population. Several mechanisms have been proposed through which parental anxiety an depressive symptoms might be related to the offspring level of psychopathology [for a review, see (15)]. Notably, several lines of research suggest that there is significant amount of genetic heritability of depression and anxiety (16). On the other hand, the role of environmental exposure has also been highlighted to explain this co-occurrence, notably the fact that parental depression or anxiety places children at increased risk of being exposed to stressful events (e.g. parental conflict), as well as negative cognitions, behaviors, and affects (17). Interestingly, research conducted on genetic syndromes and/or children with developmental disabilities (e.g. autism spectrum disorder, intellectual disability) also highlight that specific characteristics of the children might contribute to explain the emergence of anxiety and depressive symptoms in the parents (18) and that children’s symptoms and parental stress might exacerbate each other (19–22).

In summary, current evidence from the general population suggest that parental anxiety and depression and psychopathology in the offspring mutually influence each other through reciprocal interactions that are driven both by genetic and environmental mechanisms. In the current study, we explored for the first time the association between parental anxiety and depression levels and offspring psychopathology in a sample of individuals with 22q11DS and typically developing controls, using a longitudinal design. Specifically, we expected to observe significant associations between parental anxiety and depression levels and a broad spectrum of psychopathological manifestations in children, as measured with a variety of techniques (parent-reported questionnaire, self-reported questionnaire, and clinician-based semi-structured interview). Secondly and based on existing literature, we expected to observe bi-directional associations between parental anxiety and depression and offspring psychopathology. Finally, we hypothesized to observe stronger associations between parental anxiety and depression and psychopathology in the offspring affected by 22q11DS compared to typically developing offspring, thus offering supporting evidence that GxE interactions contribute to explain the heterogeneity of the clinical phenotype in this population.

## Methods

### Cohort

This study was conducted in the context of a large prospective longitudinal study on 22q11DS, which began in 2000 and has been described in previous literature [e.g. (23)]. Recruitment, which is still ongoing, was performed through patient associations and word of mouth. 22q11DS was confirmed using quantitative fluorescent polymerase chain reaction. Healthy controls (HCs) were recruited among unaffected siblings of patients (N=59) and from the Geneva State School System (N=29). Given the reduced prevalence of 22q11DS, currently estimated at 1/3,000–1/6,000 live births (24), age at recruitment varied across subjects. From an initial pool of 167 participants (271 visits) with 22q11DS and 149 HCs (261 visits), only participants aged > 11 years at the time of assessment (i.e. youngest age at which the youth self-report can be collected) with valid self- and parent-reported questionnaires and those for whom information regarding anxious-depressive symptoms, namely the Beck Anxiety Inventory [BAI (25)] and Beck Depression Inventory [BDI (26)], was available for at least one parent were included in the present study (see **Supplementary Figures S1** and **S2** for details for a flow-chart of inclusion/exclusion of participants from the entire Swiss 22q11DS longitudinal cohort). The final sample consisted of 103 (197 visits) participants with 22q11Ds and 88 HCs (129 visits). Maternal BDI and BAI were available for 101 individuals and 194 visits in 22q11DS and for 87 individuals and 129 visits in HCs. Paternal BDI and BAI were available for 88 individuals and 153 visit in 22q11DS and for 73 individuals and 105 visits in HCs.

Once recruited, both patients and HCs were followed up longitudinally every 3.7 ± 0.8 years in HCs and 3.66 ± 0.89 in 22Q11DS. Extent of longitudinal follow-up also varied from one to three visits. Specifically, among individuals with 22q11DS, 39 subjects had one visit, 34 subjects had two visits, and 30 subjects had three visits. Among HCs 55 subjects had one visit, 26 subjects had two visits and seven subjects had three visits. As detailed in the statistical analysis section, mixed models linear regression was employed to deal with the complex structure of the dataset.

Groups were matched for age at first assessment (t=0.353, p=0.73) and sex (X_2__=_ 0.032, p=0.86) and time between visits (3.68 +/− 1.49 in HCs and 3.65 ± 1 in 22q11DS, p=0.79) (see **Table 1** for the full descriptive characteristics of the sample).

**Table 1:** Demographic Table: Description and comparison of demographic features across 22q11DS and Healthy Controls samples and across sub-groups of 22q11DS and Healthy Controls divided on the basis of parental symptoms.

	22q11DS			HCs		
	Total sample	Low parental symptoms	High parental symptoms	Total sample	Low parental symptoms	High parental symptoms
Nb participants/Nb Visits	103/197	64/130	39/67	88/129	59/85	29/44
Mean Age	16.54 +/- 4.61	16.16+/- 4.11	17.17+/-5.34	16.77 +/- 4.27	16.30 +/- 3.52	17.73 +/-5.45
Male/Female	49/54	32/32	17/22	43/45	27/32	16/13
Participant living with parent(s) (% yes)	97 (94.17)	62 (96.88)	35 (89.74)	77 (87.5)	56 (94.92)	21 (72.41)
Both parents living together (% yes)	80 (78.43)^1^	47 (73.44)	33 (84.62)	71 (80.68)	43 (72.88)	28 (96.55)
Family’s annual income (%) - < 30’000Eu/year - 30’000 – 75’000 Eu/year - > 75’000Eu/year	17 (16.67) 51 (50.00) 34 (33.33)^1^	8 (12.5) 30 (46.88) 26 (40.63)	9 (23.08) 21 (53.85) 8 (21.05)^1^	11 (12.5) 35 (39.77) 42 (47.73)	2 (3.39) 27 (45.76) 30 (50.85)	9 (31.03) 8 (27.59) 12 (41.38)
Maternal level of education (%) - primary education - secondary education - higher education	12 (11.88) 34 (33.66) 55 (45.46)^2^	6 (9.38) 22 (29.73) 36 (56.26)	6 (16.22) 12 (32.43) 19 (51.35)^2^	8 (9.09) 31 (35.23) 49 (55.68)	5 (8.47) 23 (38.98) 31 (52.54)	3 (10.34) 8 (27.59) 18 (62.07)
Maternal employment (% yes)	78 (77.23)^2^	48 (76.19)^3^	30 (78.95)^3^	64 (72.73)	45 (76.27)	19 (65.52)
Paternal level of education	22 (21.78) 29 (28.71) 50 (49.50)^4^	11 (17.46) 17 (26.98) 35 (55.56)^5^	11 (28.95) 12 (31.58) 15 (39.47)^5^	18 (20.93) 22 (25.58) 46 (53.49)^9^	8 (14.04) 19 (33.33) 30 (52.63)^9^	10 (34.48) 3 (10.34) 16 (55.17)
Paternal employment (% yes)	86 (87.76)^6^	60 (96.77)^7^	26 (72.22)^8^	75 (87.21)^9^	53 (92.98)^9^	22 (75.86)
Nb Mothers/Nb Visits	101/194	64/129	37/65	87/129	58/85	29/44
Nb Fathers/Nb Visits	88/153	52/90	36/63	73/105	44/64	29/41

1 Missing information for 1 family.

2 Missing information for 2 mothers.

3 Missing information for 1 mother.

4 Missing information for 2 fathers.

5 Missing information for 1 father.

6 Missing information for 4 fathers/1 deceased.

7 Missing information for 1 father/1 deceased.

8 Missing information for 3 fathers.

9 Missing information for 2 fathers.

For participants with multiple visits, information related to the 1^st^ visit is reported in the table.

### Clinical Instruments

In both 22q11DS individuals and HCs psychopathology was firstly assessed with a combination of the Child Behavioral Checklist [CBCL (13)] before 18 years of age and the Adult Behavioral Checklist [ABCL (27)] after the age of 18, which were filled out by parents, considering the “total problems”, “internalizing problems”, and “externalizing problems” scales. Additionally, we employed the Youth Self Report Questionnaire [YSR (13)] before 18 years of age and the Adult Self Report Questionnaire [ASR (27)], which was filled out directly by both individuals with 22q11DS and HCs, again considering “total problems”, “internalizing problems”, and “externalizing problems” scales. In *post-hoc* analyses, the syndrome scales (anxious-depressed symptoms, somatic concerns, thought problems, rule-breaking behavior, aggressiveness, withdrawn symptoms, and attention problems) of the CBCL/ABCL and YSR/ASR were also used. Moreover, we used the Structured Interview for Psychosis-Risk Syndromes [SIPS (28)] only in individuals with 22q11DS, considering the mean positive, negative, disorganized, and generalized symptoms subscales.

Parental anxious-depressive psychopathology was assessed with a combination of the BDI total score [BDI (26)] and BAI total score [BAI (25)]. In order to obtain an average measure of anxious-depressive psychopathology, total BDI and BAI scores were firstly separately z-scored and then averaged for each parent. For visits for which both maternal and paternal psychopathology measures were available, these were averaged across parents in order to obtain an overall estimated of parental anxious-depressive psychopathology (a comparison between maternal and paternal symptoms is described in the **Supplementary Analyses A** and **Supplementary Figure S3**).

### Statistical Analyses

We employed mixed-models linear regression (MMLR), to characterize and test the effects of parental anxious-depressive psychopathology in developmental trajectories of child psychopathology, separately in HCs and 22q11DS. Specifically, samples of 22q11DS individuals and HCs were each separately divided in two sub-samples, according to whether parental anxious-depressive psychopathology at the earliest available visit, was higher or lower that the observed mean parental psychopathology. This procedure yielded four samples: high-parental-psychopathology HCs (29 subjects, 44 visits, mean-age 18.6+/−5.3, male/female 13/16) low-parental-psychopathology HCs (59 subjects, 87 visits, mean-age 17.4+/−4.0, male/female 32/27), high-parental-psychopathology 22q11DS (39 subjects, 68 visits, mean-age 19.7+/−5.9, male/female 17/22), and low-parental-psychopathology 22q11DS (64 subjects, 130 visits, mean-age 18.6+/−4.4, male/female 32/32). Sub-samples were matched for age (P=0.13 in 22q11DS, P=0.13 in HCs) and gender (P=0.53 in 22q11DS, P=0.41 in HCs).

We then employed MMLR to compare developmental trajectories of child psychopathology between high vs low parental psychopathology sub-samples in 22q11DS and HCs separately. Indeed, MMLR is ideally suited for longitudinal samples with variable number of visits across subjects and ariable age at the first assessment and variable interval between visits (29). Our approach, has been detailed in previous publications from our group (29). Briefly, developmental trajectories for each psychopathological measure we estimated by fitting random slope models of increasing order, from constant to cubic, to the association between age and each variable being tested. Population parameters (age and diagnosis) were modeled as fixed effects and within-subject factors as random effects by using the nlmfit function implemented in MATLAB_R2018a (Mathworks). Subsequently, the Bayesian information criterion (BIC) was employed to select the optimal model order, while avoiding over-fitting. Finally, we applied a likelihood ratio test to evaluate differences in trajectories between groups both in terms of shape differences (curves that do not follow a parallel path for both groups) and intercept differences (curves that follow parallel paths in both groups, but at different intercepts).

As a subsequent analysis, we were interested in comparing the strength the association between child and parental psychopathology across 22q11DS and HCs, in order to test for the existence of potential GxG or GxE interactions. Hence, we computed Pearson correlations between mean parental psychopathology and child total CBCL and YSR scores, which were averaged across multiple visits for each subject, separately for HCs and 22q11DS samples. We then performed Fisher’s R to Z transform to test for differences in the strength of the parental-child psychopathology correlation across samples.

As a final analysis were employed our longitudinal sample to attempt to discern the causal directionality of the association between parental and child psychopathology. Hence, we restricted our analysis to families for whom measures of child and parental psychopathology were available for at the least two longitudinal assessments (N=64, mean age at baseline 16.8+/−4.0, mean age at follow-up 20.6+/−4.19). We then computed both the longitudinal association between baseline child psychopathology and parental psychopathology at follow-up and the reverse association between baseline parental psychopathology and subsequent child psychopathology scores at follow-up. Finally, we computed parent-child longitudinal correlation after accounting for the effects of homologous correlations between child-baseline to child-follow-up psychopathology and parent-baseline to parent-follow-up psychopathology, using partial correlations.

## Results

### Trajectories of Child Psychopathology in High vs Low Parental Psychopathology Sub-Groups

All measures of child psychopathology were stable across age, as indicated by the choice of a constant model order 0, in the mixed model linear regression.

**Figure 1:**
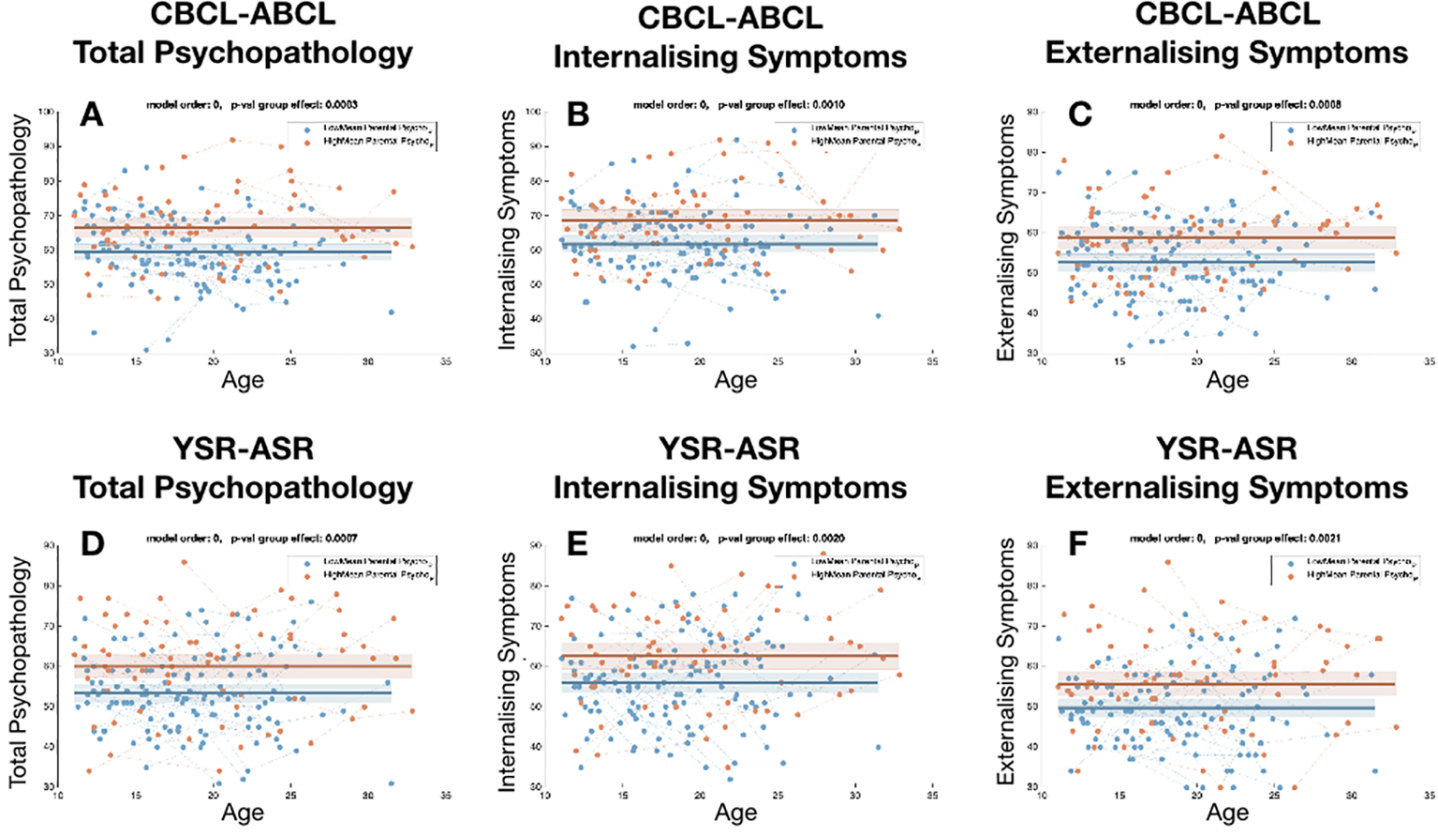
Comparison of the trajectories of child psychopathology across sub-group of 22q11DS individuals, divided according to levels of parental psychopathology. **(A)** Total psychopathology score for CBCL-ABCL. **(B)** Internalizing symptom score for CBCL/ABCL, **(C)** Externalizing symptom score for CBCL/ABCL. **(D)** Total psychopathology score for YSR-ASR. **(E)** Internalizing symptom score for YSR-ASR, **(F)** Externalizing symptom score for YSR-ASR.

In the 22q11DS sample, children in the high parental psychopathology subgroup presented significantly higher levels of total psychopathology compared to the low parental psychopathology subgroup irrespectively of age, as measured by both the CBCL total score (p=0.0003) and YSR total score (P=0.0007). Such effects were driven by significantly increased levels of both internalizing symptoms (CBCL-internalizing P=0.001, YSR-internalizing P=0.002) and externalizing symptoms (CBCL-externalizing P=0.0008, YSR-externalizing P=0.002) in 22q11DS individuals with high parental psychopathology (see **Figure 1**). As a *post-hoc* analysis, we compared trajectories for individual items of the CBCL-ABCL and YSR-ASR questionnaires. We observed significantly higher scores in the high-parental psychopathology sub-group in most examined items for both YSR-ASR and CBCL-ABCL questionnaires including anxious-depressive score (P-YSR=0.0017, P-CBCL= 0.0012), somatic concerns (P-YSR=0.041, P-CBCL= 0.0011), thought problems (P-YSR=0.0043, P-CBCL= 0.014), rule-breaking behavior (P-YSR=0.0033, P-CBCL= 0.0021), and aggressiveness (P-YSR=0.0007, P-CBCL= 0.0011). Withdrawn symptoms were higher in the high parental psychopathology group only when considering the YSR-ASR (P-YSR=0.01, P-CBCL= 0.187) as were attention problems (P-YSR=0.025, P-CBCL= 0.125). A comparison between the specific effect of maternal vs. paternal psychopathology is described in **Supplementary Analyses B** and **Supplementary Figures S4–S6**.

When examining effects on SIPS sub-scores we did not observe significant differences for SIPS disorganized sub-scale (P=0.11) and only a non-significant trend-level increase in SIPS positive symptom subscale in the high-parental-psychopathology subgroup (P=0.055). However, 22q11DS individuals with high parental psychopathology presented significantly higher levels of SIPS negative symptoms (P=0.03) and SIPS generalized symptoms (P=0.02) compared to the low parental psychopathology sub-group. *Post-hoc* analyses revealed that differences in negative symptoms were driven by more impaired ideational richness (P=0.03) and occupational functioning (P=0.02), whereas differences in the generalized symptoms subscale were driven by more severe dysphoric mood (P=0.006), in the high parental psychopathology sub-group (results for SIPS subscores are presented in **Supplementary Figure S7**).

**Figure 2:**
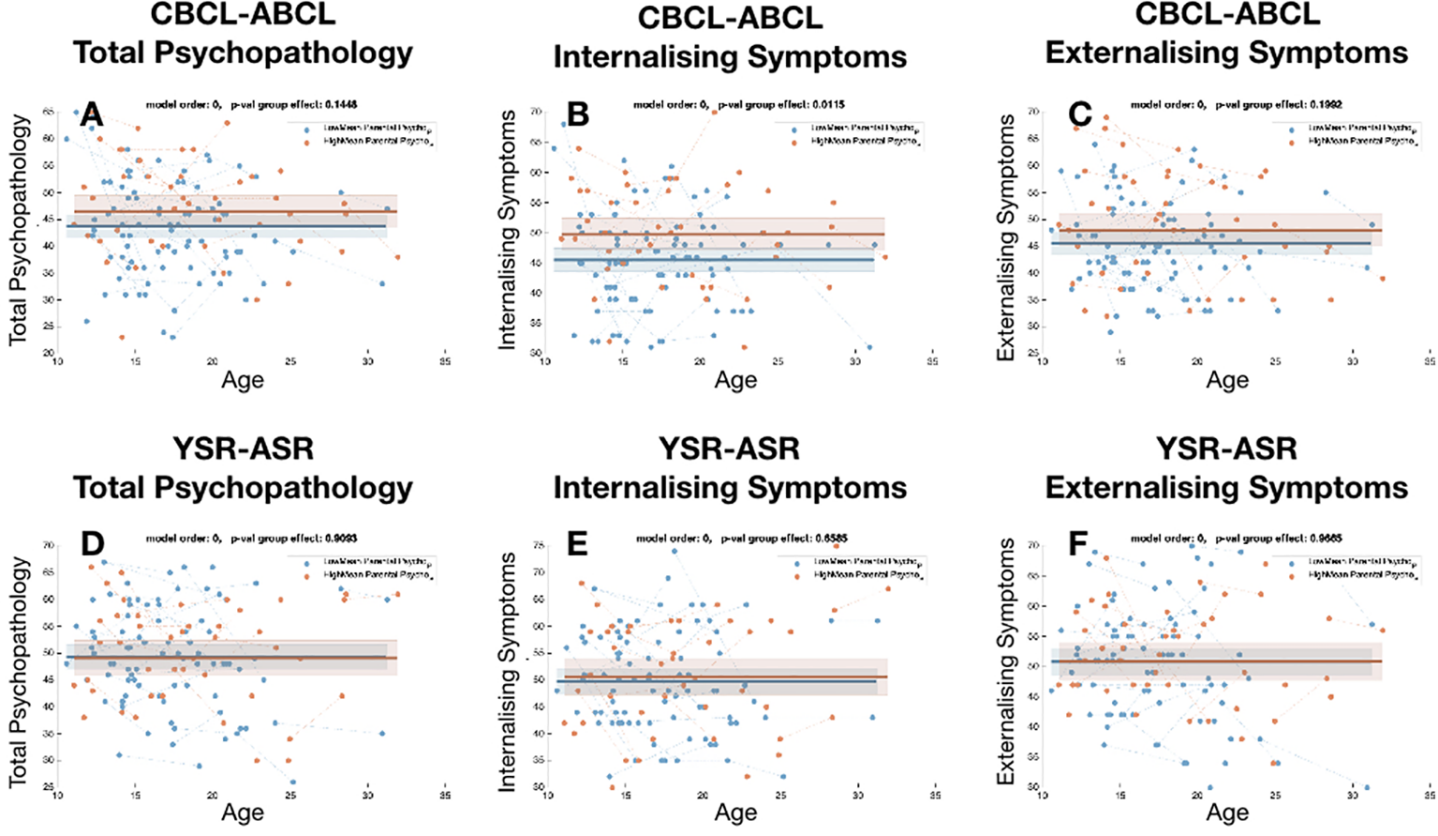
Comparison of the trajectories of child psychopathology across sub-group of healthy controls, divided according to levels of parental psychopathology. **(A)** Total psychopathology score for CBCL-ABCL. **(B)** Internalizing symptom score for CBCL/ABCL, **(C)** Externalizing symptom score for CBCL/ABCL. **(D)** Total psychopathology score for YSR-ASR. **(E)** Internalizing symptom score for YSR-ASR, **(F)** Externalizing symptom score for YSR-ASR.

When dividing HCs according to levels of parental psychopathology we did not observe significant difference in levels overall child psychopathology as estimated by both CBCL total score (P=0.144) and YSR total score (P=0.9). Difference were similarly not significant for externalizing symptoms (CBCL-externalizing P=0.19, YSR-externalizing P=0.96). We only observed significantly higher levels of CBCL internalizing scores in HCs with high parental psychopathology (P=0.01) that was however not replicated for YSR internalizing score (P=0.65) (see **Figure 2**).

A summary of the comparisons between subgroups of individuals with 22q11DS and HCs divided according to levels of parental psychopathology can be found in the **Supplementary Table S1**.

### Comparison of the Association of Child and Parental Psychopathology Across 22q11DS and HCs

**Figure 3:**
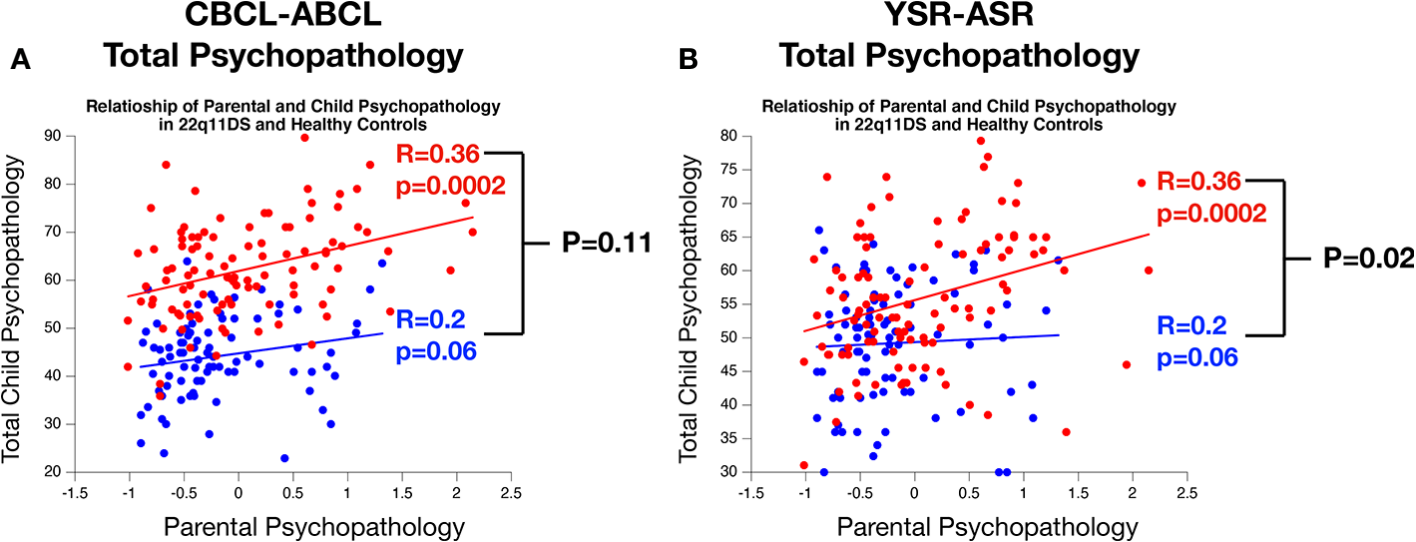
Comparison of the association of parental and child psychopathology in 22q11DS and healthy controls. **(A)** Comparison of the association of parental psychopathology with CBCL-ABCL total psychopathology score. **(B)** Comparison of the association of parental psychopathology with YSR-ASR total psychopathology score.

In accordance with previous results in 22q11DS we observed a significant linear association between mean measures of child and parental psychopathology for both CBCL-total-score (R=0.36, p=0.0002) and YSR total score (R=0.31, p=0.001). Correlations between child and parental psychopathology were not significant in HCs for CBCL-total-score (R=0.2, p=0.06) or YSR-total-score (R=0.04, P=0.65) (see **Figure 3**).

A direct comparison of association strength across samples revealed that the CBCL-total score correlation with parental psychopathology was not significantly different across sample (R=0.36 in 22q11DS vs R=0.2 in HCs, P=0.11). However in 22q11DS, we observed a significantly stronger association between parental psychopathology and child YSR-total score compared to HCs (R= 0.36 vs R=0.04, P=0.02) (see **Figure 3**).

### Longitudinal Bi-Directional Associations Between Child and Parental Psychopathology in 22q11DS

**Figure 4:**
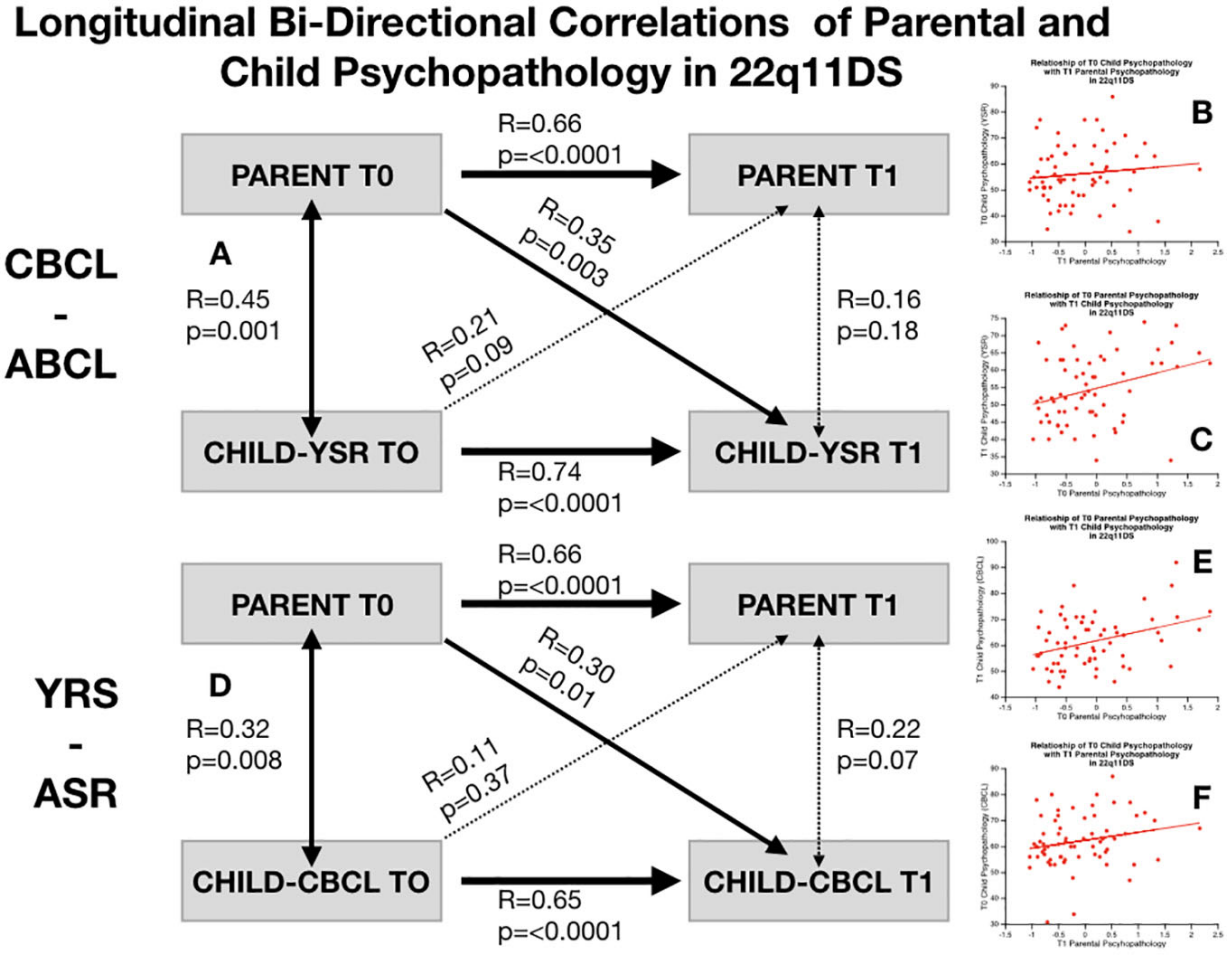
Structure of longitudinal bi-directional correlations of parental and child psychopathology in 22q11DS. **(A)** Structure of longitudinal correlations between parental psychopathology and child CBCL-ABCL total psychopathology score. Bold arrows indicated statistically significant correlations whereas dashed arrows indicate non-statistically significant correlations. **(B)** Correlation of baseline child CBCL-ABCL total psychopathology score with parental psychopathology at follow-up. **(C)** Correlation of parental psychopathology at baseline with child CBCL-ABCL total psychopathology at follow-up. **(D)** Structure of longitudinal correlations between parental psychopathology and child YSR-ASR total psychopathology score. Bold arrows indicated statistically significant correlations whereas dashed arrows indicate non-statistically significant correlations. **(E)** Correlation of baseline child YSR-ASR total psychopathology score with parental psychopathology at follow-up. **(F)** Correlation of parental psychopathology at baseline with child YSR-ASR total psychopathology at follow-up.

**Figure 5:**
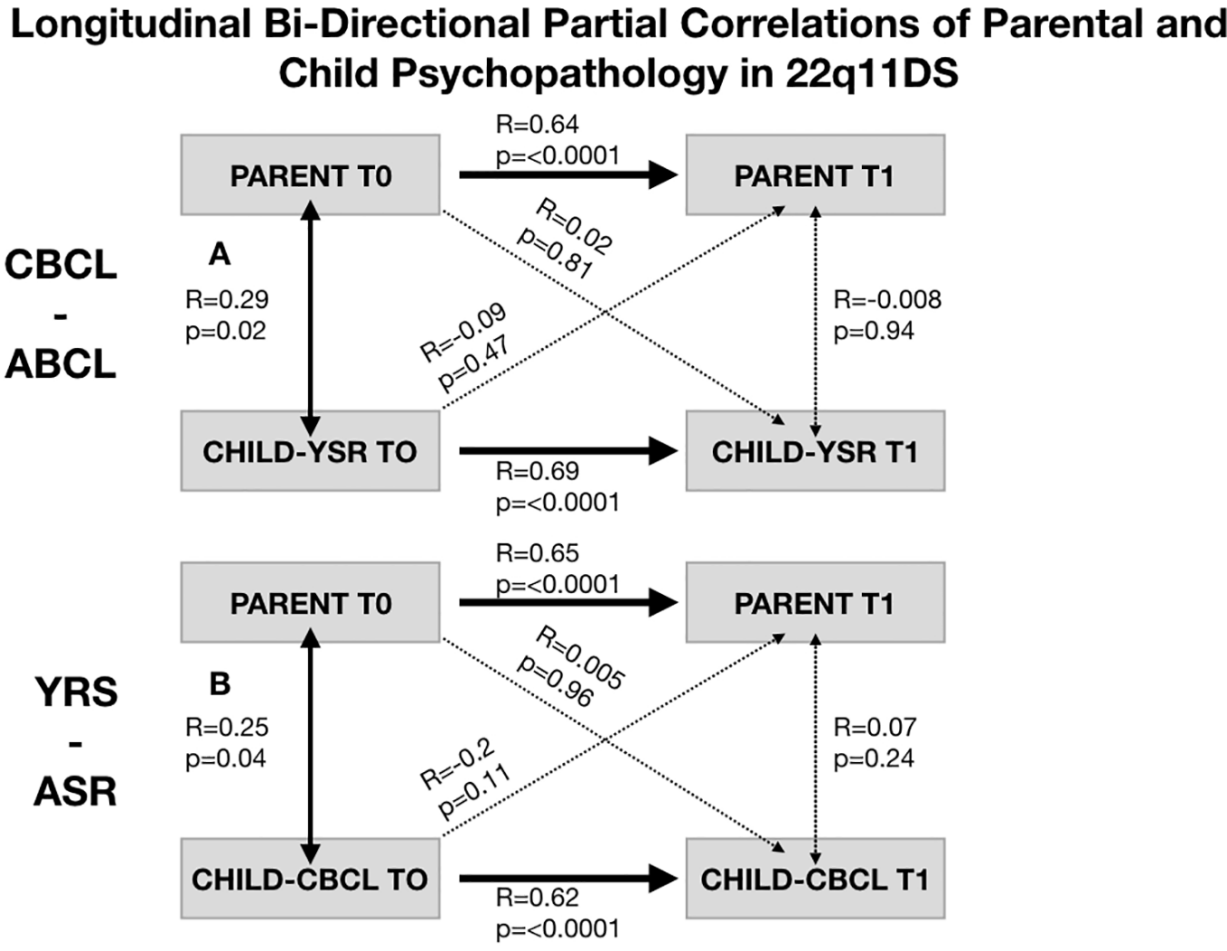
Structure of longitudinal bi-directional partial correlations of parental and child psychopathology in 22q11DS. **(A)** Structure of longitudinal partial correlations between parental psychopathology and child CBCL-ABCL total psychopathology score. Bold arrows indicated statistically significant correlations whereas dashed arrows indicate non-statistically significant correlations. **(B)** Structure of longitudinal partial correlations between parental psychopathology and child YSR-ASR total psychopathology score. Bold arrows indicated statistically significant correlations whereas dashed arrows indicate non-statistically significant correlations.

In 22q11DS we observed a significant association between parental psychopathology at baseline and child YSR psychopathology at follow-up (R-YSR=0.3, P=0.01, R-CBCL=0.35, P=0.003). On the other hand, child psychopathology at baseline did not significantly predict parental psychopathology at follow-up (R-YSR=0.11, P=0.37, R-CBCL=0.21, P=0.09) (see **Figure 4**). However, when we computed partial correlations considering the effects of homologous correlation between Child-baseline to child follow-up and parent-baseline to parent-follow-up, parent-child longitudinal correlations were no longer significant (R-YSR= 0.005, p=0.96, R-CBCL=0.02, P=0.81) (see **Figure 5**).

## Discussion

The present study aimed to examine the association between parental levels of anxiety and depression symptoms and psychopathology in their offspring with 22q11DS as well as typically developing controls. Overall, we found significant associations between the two constructs in the 22q11DS group, with parental level of anxiety and depression being related to a widespread increase of psychopathological manifestations measured with both self- and parent-reported questionnaires. Interestingly, associations were significantly stronger in the 22q11DS population compared to the HC group. When considering purely longitudinal analyses in the 22q11DS group, we observed a significant association between parental anxiety and depression level at baseline and offspring psychopathology at follow-up, whereas the opposite association was statistically not significant. However, we observed an overall stability of both parental and child psychopathology across longitudinal assessments, leading to strong homologous associations.

The main result of the present study is that higher levels of parental anxious-depressive symptoms are related to the level of psychopathology, specifically in their offspring with 22q11DS. These associations were observed for both internalizing and externalizing dimensions and almost all the CBCL/ABCL and YSR/ASR subscales. That being said, one of the strongest associations was with the anxious-depressed dimension of both the CBCL/ABCL and YSR/ASR. A strength of this study was the simultaneous use of both self- and parent-reported instruments, leading to comparable results. This allows us to discard the fact that those results could be driven by a biased assessment of the offspring’s psychopathology by more anxious or depressed parents. Significant associations were also found between parental anxiety and depressive level and the SIPS negative and general dimensions. The results for the SIPS general dimension were mostly driven by an effect of parental anxiety and depression on the offspring’s dysphoric mood, which is in line with results obtained with the self- and parent reported questionnaires. Altogether, these results highlight that the strongest associations between parent and offspring with 22q11DS were observed for homologous associations between the affective dimension of psychopathology, similar to what has been described in the general population (15). Decreased occupational functioning (SIPS N6) and impaired ideational richness (SIPS N5) mostly accounted for the results for the SIPS negative dimension. In a previous study by our group (30), those two items were found to load on a different dimension compared to the remaining SIPS negative items, suggesting that they rather reflect aspects of the 22q11DS clinical phenotype that are less directly related to psychosis. On the other hand, “core” negative symptoms of psychosis were found to be not significantly related to parental anxiety and depression. In line with this observation, we found only trend-level associations with the SIPS positive dimension and no effect with the disorganized dimension. This suggests that there is no direct link between parental anxiety and depression and psychosis in 22q11DS.

Several complementary interpretations can be put forth to account for these associations. An important aspect pertains to the directionality of the association. In the present study, we exploited the longitudinal nature of the data to attempt to address this question of directionality. Overall, we detected a strong stability of both parental and child psychopathology across time and observed that cross sectional correlations of parental and child psychopathology were significantly stronger than longitudinal ones. These results might suggest the existence of bi-directional influences occurring in a short time frame. Indeed, this is highly likely that the long interval between baseline and follow-up assessments (i.e. 3.5 years) did not allow to fully grasp the directionality of complex and dynamic interactions between child and parent psychopathology. Still, we found that parental level of anxiety and depression at baseline predicted offspring psychopathology at follow-up but that the reverse direction was not significant. While such longitudinal correlations were no longer significant after accounting for baseline child psychopathology, these results provide some provisional evidence for a higher sensibility of offspring with 22q11DS to parental psychopathology rather than an opposite parental sensibility to the level of their offspring psychopathology.

In the general population, a large body of literature shows that specific characteristics of the family environment can act either as resilience or risk factors in vulnerable populations. In particular, research indicates that parental anxiety or depression increase the risk of psychopathology in the offspring through complex genetic and environmental mechanisms [for a review, see (15)]. Notably, classical heritability studies show that genetic factors play a significant role regarding the transmission of psychopathology across generations (16). These results have been confirmed using modern Genome Wide Association Studies (GWAS) approaches demonstrating the role of polygenic heritability mechanisms, which have been operationalized by, for instance, polygenic risk scores for depression (31). On the other hand, the role of environmental factors have also been highlighted, notably the fact that parental depression or anxiety places children at increased risk of being exposed to stressful events (e.g. parental conflict), as well as negative cognitions, behaviors, and affects (17). In this regard, probably the most interesting finding from the present study is that the strength of the association between parental anxiety and depression level and offspring psychopathology was significantly stronger in the 22q11DS group compared to typically developing controls. Indeed, this provides at least provisional evidence for the existence of GxG and/or GxE interactions contributing to the emergence of psychopathology in individuals with 22q11DS. As pertains to GxG interactions, recent studies have shown that genetic variants outside of the 22q11.2 locus can significantly modulate the risk for schizophrenia in this population (6). Our results potentially suggest that similar mechanisms could occur for other dimensions of psychopathology. This hypothesis could be explicitly tested using polygenic risk score approaches for affective disturbances. Regarding GxE interactions, recent evidence in 22q11DS has suggested that at least part of the genetic vulnerability to psychopathology—including both psychotic and non-psychotic manifestations—may be mediated by increased vulnerability environmental factors, such as exposure to stressful life events (9).

As mentioned previously, we found no direct link between parental anxiety and depression and psychosis in the sample of participants with 22q11DS, but broad effects on several forms of non-psychotic symptomatology spreading across both internalizing and externalizing dimensions. Recent findings in individuals at clinical high-risk for psychosis (without 22q11DS) have recognized the high prevalence of non-psychotic manifestations, including notably anxiety and depression (32). It is becoming increasingly clear that especially in the early phases, such non-specific manifestation play a role in the pathway toward psychosis and, in general, more severe functional impairments (33). These observations have been conceptualized as either evidence for the existence of common etiology mechanisms shared across all forms of psychopathology (34) or the evidence of the causal role of affective disturbances in the pathway toward psychosis (35). In line with this, the presence of an anxiety disorder has been shown as a significant risk factor for the emergence of psychotic disorders in 22q11DS (36). Altogether, our findings suggest that parental anxiety and depression could be an important upstream variable in the cascade toward more severe forms of psychopathology in this genetically vulnerable group, through a broad increase of non-psychotic manifestations.

From a clinical point of view, these results highlight several considerations. Firstly, it highlights the fact that the conceptualization of psychopathology in 22q11DS should be understood through an integrative approach that also takes the level of parental psychological well-being into account. Indeed, our findings could suggest that despite a strong genetic predisposition, vulnerability to psychopathology in 22q11DS can still be modulated protective environmental factors such as parental well-being. Critically, this implies that parents should not be left alone with the psychological burden of raising a child with 22q11DS. At the practical level, it suggests a rationale for a systematic assessment of parental well-being in the context of 22q11DS. This could be operationalized as a two-step assessment, including a systematic screening and a more in-depth investigation with parents for whom concerns were raised. The management of parental well-being should begin in the very first stages, notably in the communication of the 22q11DS diagnosis. Indeed, several studies have highlighted a more negative diagnosis experience in families of patients with 22q11DS compared to other genetic conditions (37), which could eventually lead to increased stress and the development of (subthreshold) anxiety and depression. In case of concerns regarding parental well-being, a staged approach could be envisioned, starting from family psycho-education about 22q11DS, to practical measures aimed at alleviating parental burden, and extending to family psychotherapeutic interventions.

The present study should be interpreted in light of the following limitations. Firstly, the assessment of parental symptoms only consisted of self-reported measures and focused only on anxiety and depressive symptoms. Secondly, whereas the longitudinal nature of the study can be considered as a strength, the time interval between the two visits was too long to fully address the issue of directionality of the effects between parental and offspring’s symptoms. This could be better addressed in future studies using repeated measures occurring at high temporal resolution, for example through the implementation of experience sampling methodology (ESM) protocols. Finally, the design of the study did not allow to disentangle the role of genetic vs. environmental mechanisms linking levels of psychopathology across generations.

## Conclusion

In conclusion, we found significant associations between parental levels of anxiety and depression and level of psychopathology in offspring with 22q11DS. This highlights that the conceptualization of psychopathology in 22q11DS should be understood through an integrative approach that also takes the level of parental psychological well-being into account.

## Data Availability Statement

The raw data supporting the conclusions of this article will be made available by the authors, without undue reservation.

## Ethics Statement

The studies involving human participants were reviewed and approved by Geneva Ethic Committee. Written informed consent to participate in this study was provided by the participants’ legal guardian/next of kin.

## Author Contributions

MA and SE have designed the study and written the manuscript. CS and MS have conducted the statistical analysis and written the manuscript.

## Funding

This study was supported by a grant from the Swiss National Science Foundation (SNSF) to Prof. Eliez (# 324730_144260) and The National Center of Competence in Research (NCCR) “Synapsy—The Synaptic Bases of Mental Diseases” (# 51NF40-185897). MS is supported by an Ambizione grant from the Swiss National Science Foundation (#PZ00P1_174206).

## Conflict of Interest

The authors declare that the research was conducted in the absence of any commercial or financial relationships that could be construed as a potential conflict of interest.
