# Supplementary material for: Association Between Parental Anxiety and Depression Level and Psychopathological Symptoms in Offspring With 22q11.2 Deletion Syndrome
Source: Front Psychiatry. 2020 Jul 23;11:646. doi: 10.3389/fpsyt.2020.00646 (PMC7390875; doi:10.3389/fpsyt.2020.00646)
Supplement: Supplementary file 1 [file DataSheet_1.docx]

**Supplementary Figure 1**


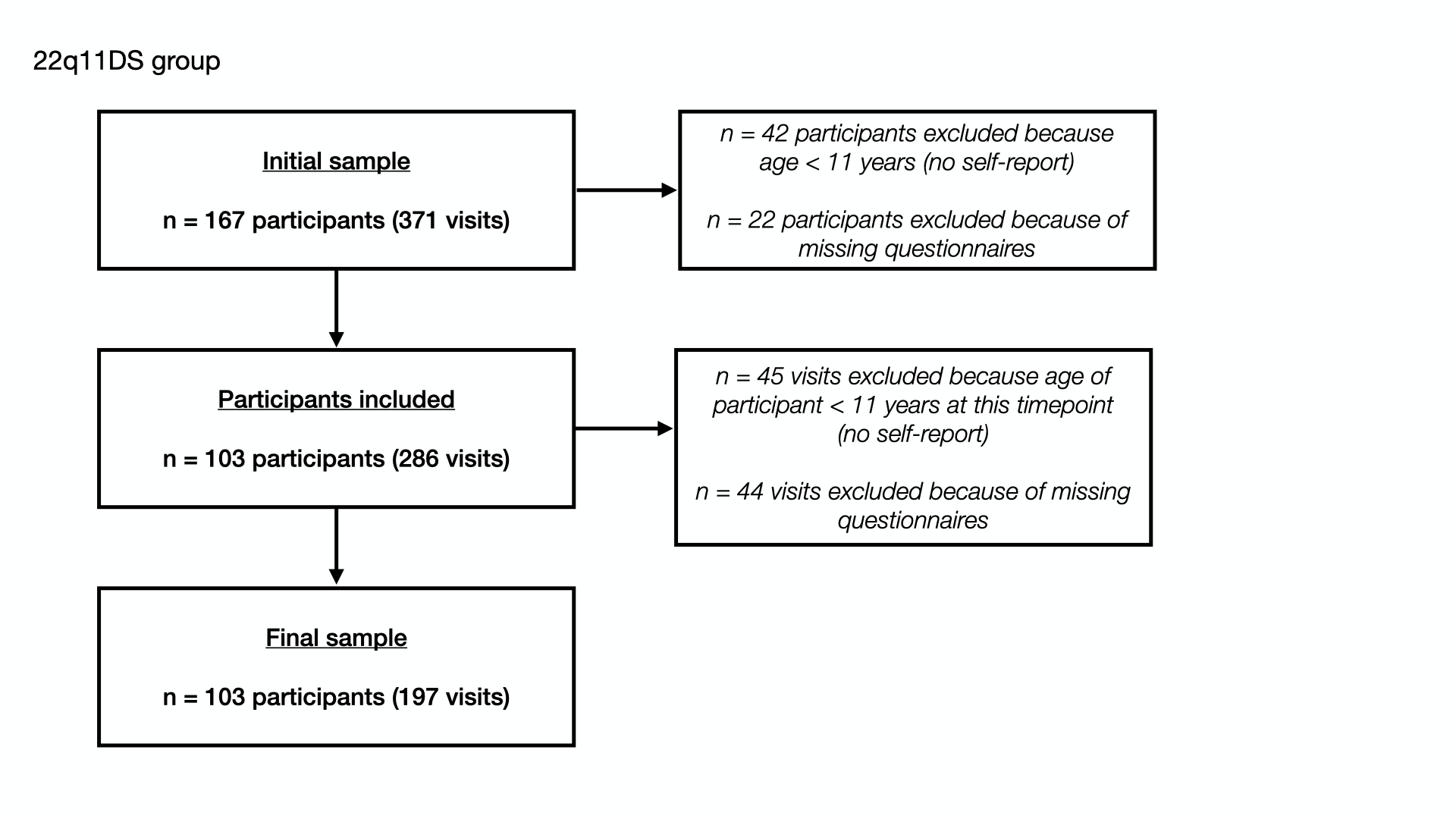


**Supplementary Figure 1:** Flow chart for selection of final sample of healthy controls included in the study.

**Supplementary Figure 2
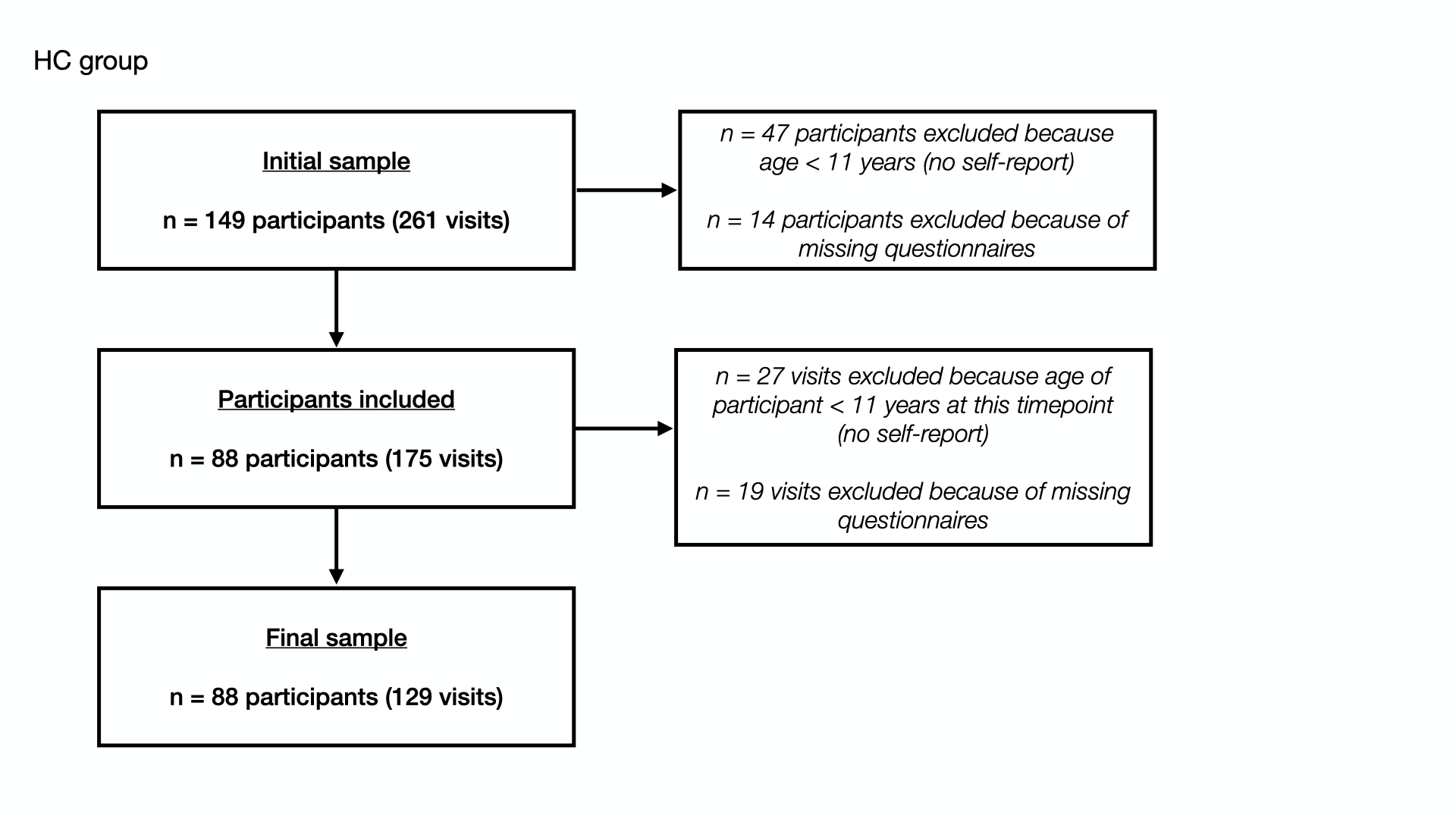
 Supplementary Figure 2:** Flow chart for selection of final sample of 22q11DS individuals included in the study.

**Supplementary Table 1**

|  | 22q11DS  High vs Low Parental Psychopathology  P-Value of Group Effect | HCs  High vs Low Parental Psychopathology  P-Value of Group Effect |
| --- | --- | --- |
| CBCL-ABCL Total Psychopathology | **P=0.0003** | P=0.144 |
| CBCL-ABCL Internalizing Symptoms | **P=0.001** | **P=0.011** |
| CBCL-ABCL Externalizing Symptoms | **P=0.0006** | P=0.199 |
| YSR-ASR Total Psychopathology | **P=0.0007** | P=0.909 |
| YSR-ASR Internalizing Symptoms | **P=0.002** | P=0.658 |
| YSR-ASR Externalizing Symptoms | **P=0.002** | P=0.965 |
| SIPS Positive Symptoms | P=0.055 | ---------- |
| SIPS Negative Symptoms | **P=0.035** | ---------- |
| SIPS Disorganized Symptoms | P=0.117 | ---------- |
| SIPS Generalized Symptoms | **P=0.026** | ---------- |

**Supplementary Table 1:** Summary table of results of comparisons of psychopathology between subgroups of individuals with 22q11DS and Healthy Controls divided according to levels of parental psychopathology.

**Supplementary Analyses A:** Comparison of maternal and paternal psychopathology in parents of children with 22q11.2DS.

**Methods:** In order to paternal vs maternal psychopathology, we firstly restricted our analyses to visits in which assessments for both parents were available (88/103 subjects 151/198 visits). We then compared levels of anxiety and depression between mothers and fathers of children with 22q11DS using mixed-models-linear-regression.

**Results:** This analysis revealed that while levels of maternal and paternal depression were comparable (p=0.138), mothers of children with 22q11DS presented significantly higher levels of anxiety compared to fathers (p=0.02).

**Supplementary Figure 3:**


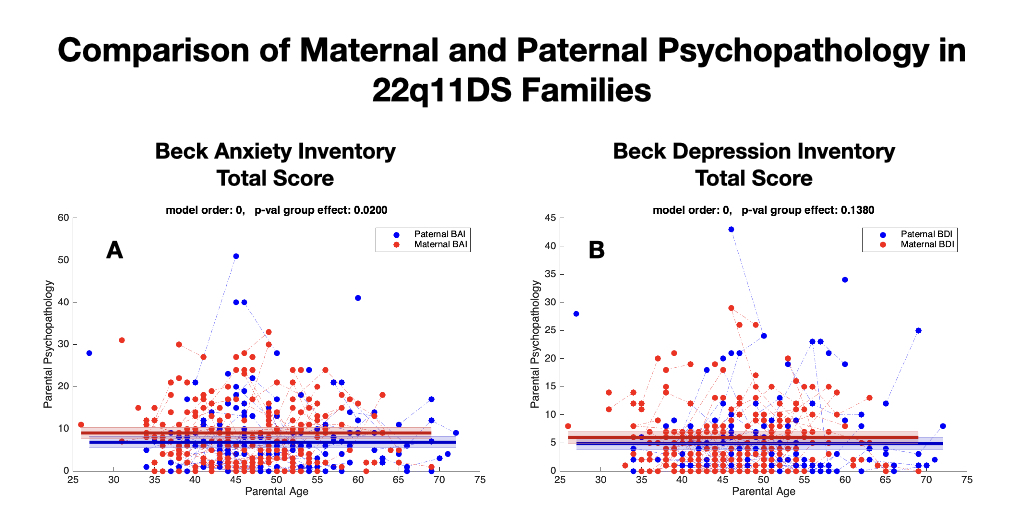


**Supplementary Figure 3:** Comparison of maternal and paternal psychopathology in parents of children with 22q11DS. **A:** Comparison of Beck Anxiety Inventory total score between mothers and fathers of children with 22q11DS**. B:** Comparison of Beck Depression Inventory total score between mothers and fathers of children with 22q11DS**.**

**Supplementary Analyses B:** Dissecting the differential associations of maternal and paternal anxiety-depression with psychopathology of children with 22q11DS.

**Methods**:

In order to compare associations with paternal vs maternal psychopathology we firstly restricted our analyses to visits in which assessments for both parents were available (85/103 subjects 147/198 visits). We then firstly divided offspring with 22q11DS purely according to levels of maternal psychopathology yielding a subgroup with high maternal psychopathology (33-subjects 60 visits, mean age = 19.97+-6) and subgroup with low maternal psychopathology (52-subjects 87 visits x-males, mean age = 17.6+-4.3). Next we repeated the procedure considering only paternal psychopathology yielding a subgroup with high paternal psychopathology (28 subjects 47 visits, mean age = 18.9+-5.7) and subgroup with low paternal psychopathology (57-subjects 100 visits x-males, mean age = 18.4+-4.9). Finally we considered the combined effect of both paternal and maternal psychopathology, in the sub-sample of patients for whom both parental assessments were available, yielding a subgroup with high parental psychopathology ( 34 subjects 59 visits, mean age = 19.9+-6.1) and subgroup with low parental psychopathology (51 subjects 88 visits , mean age = 17.9+-4.5). We compared such sub-groups in terms of both CBCL-ABCL and YSR-ASR total psychopathology and internalizing, externalizing psychopathology subscales.

**Results:**

This analysis revealed that maternal psychopathology alone was not significantly associated with CBCL-ABCL total score (p=0.152), CBCL-ABCL internalizing (p=0.158) or CBCL-ABCL externalizing (p=0.061). Moreover maternal psychopathology was not associated with YSR-ASR total score (p=0.216), or internalizing (p=0.104) and externalizing psychopathology (p=0.104) sub-scores. Paternal psychopathology was also not significantly associated with CBCL-ABCL total score (0.068) or internalizing (p=0.114), but was significantly associated with higher CBCL-ABCL externalizing psychopathology (p=0.03). However paternal psychopathology was however significantly associated with both YSR-ASR total psychopathology score (p=0.0029) as well as externalizing (p=0.0027) and internalizing psychopathology subscales (p=0.0027) . When we considered both maternal and paternal psychopathology, we observed significant strong associations with all clinical scales including CBCL-ABCL total psychopathology (p=0.0003), CBCL-ABCL internalizing (p=0.001) and CBCL-ABCL externalizing (p=0.0008) as well as with YSR-ASR total psychopathology (p=0.0007) and internalizing (p=0.002) and externalizing subscales (p=0.002). These analyses would suggest that both maternal and paternal psychopathology measures contribute to the observed association with 22q11DS child psychopathology, albeit with stronger associations between paternal psychopathology and offspring YSR-ASR measures.

**Supplementary Figure 4:**


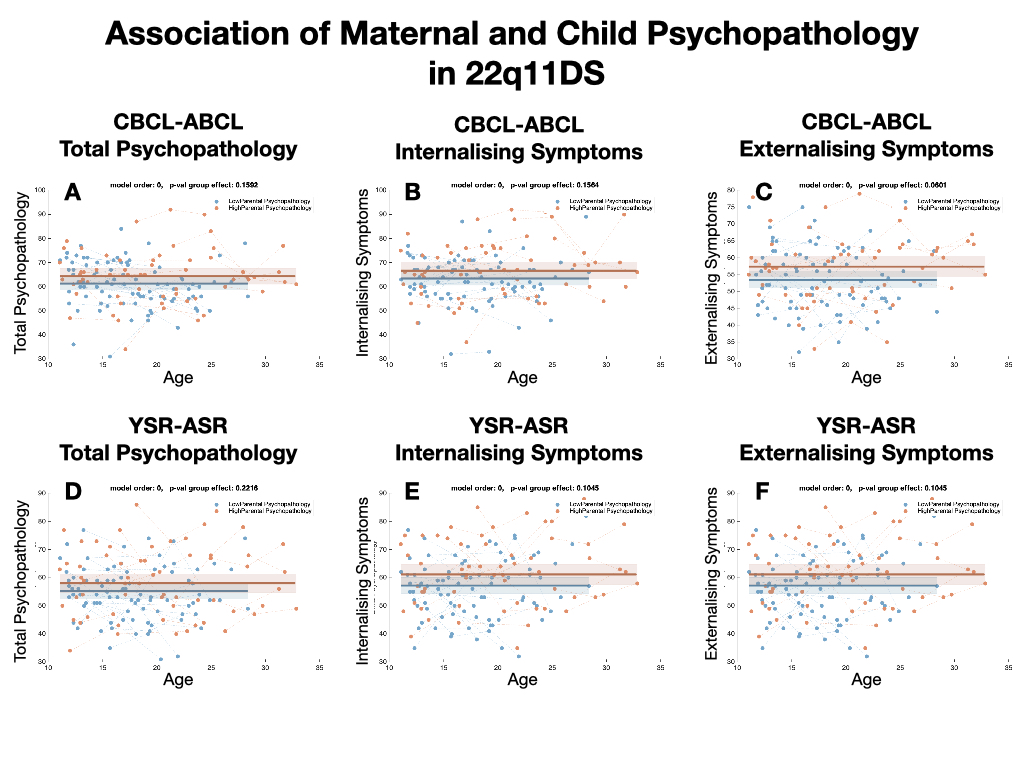


**Supplementary Figure 4:** Comparison of the trajectories of child psychopathology across sub-group of 22q11DS individuals, divided according to levels of maternal psychopathology. **A:** Total psychopathology score for CBCL-ABCL**. B:** Internalizing symptom score for CBCL/ABCL, **C:** Externalizing symptom score for CBCL/ABCL. **D:** Total psychopathology score for YSR-ASR . **E:** Internalizing symptom score for YSR-ASR, **F:** Externalizing symptom score for YSR-ASR

Supplementary Figure 5:


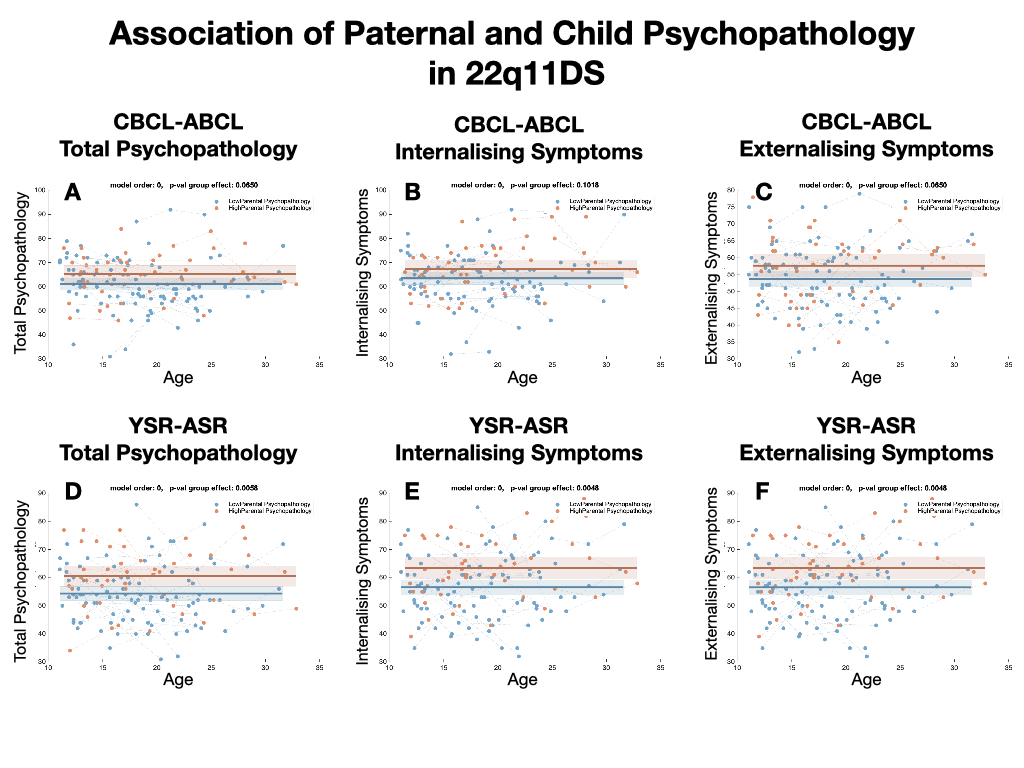


**Supplementary Figure 5:** Comparison of the trajectories of child psychopathology across sub-group of 22q11DS individuals, divided according to levels of paternal psychopathology. **A:** Total psychopathology score for CBCL-ABCL**. B:** Internalizing symptom score for CBCL/ABCL, **C:** Externalizing symptom score for CBCL/ABCL. **D:** Total psychopathology score for YSR-ASR . **E:** Internalizing symptom score for YSR-ASR, **F:** Externalizing symptom score for YSR-ASR

**Supplementary Figure 6:**


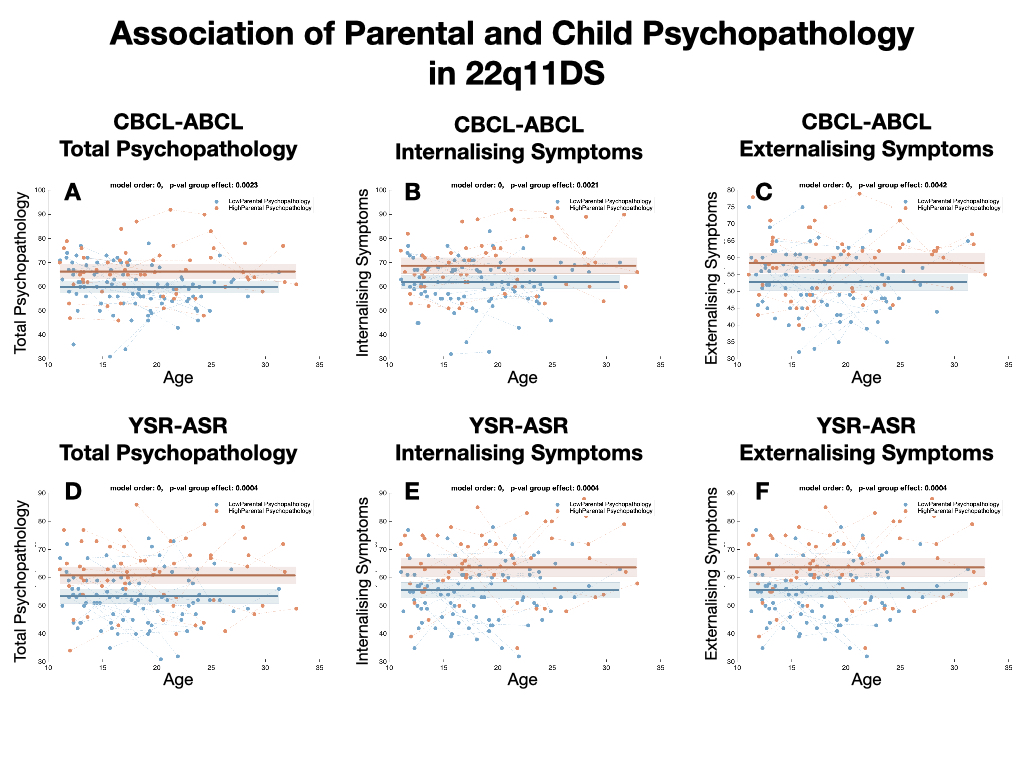


**Supplementary Figure 6:** Comparison of the trajectories of child psychopathology across sub-group of 22q11DS individuals, divided according to levels of parental psychopathology, restricting the analysis two individuals for whom both paternal and maternal assessments were available. **A:** Total psychopathology score for CBCL-ABCL**. B:** Internalizing symptom score for CBCL/ABCL, **C:** Externalizing symptom score for CBCL/ABCL. **D:** Total psychopathology score for YSR-ASR . **E:** Internalizing symptom score for YSR-ASR, **F:** Externalizing symptom score for YSR-ASR

**Supplementary Figure 7**

**
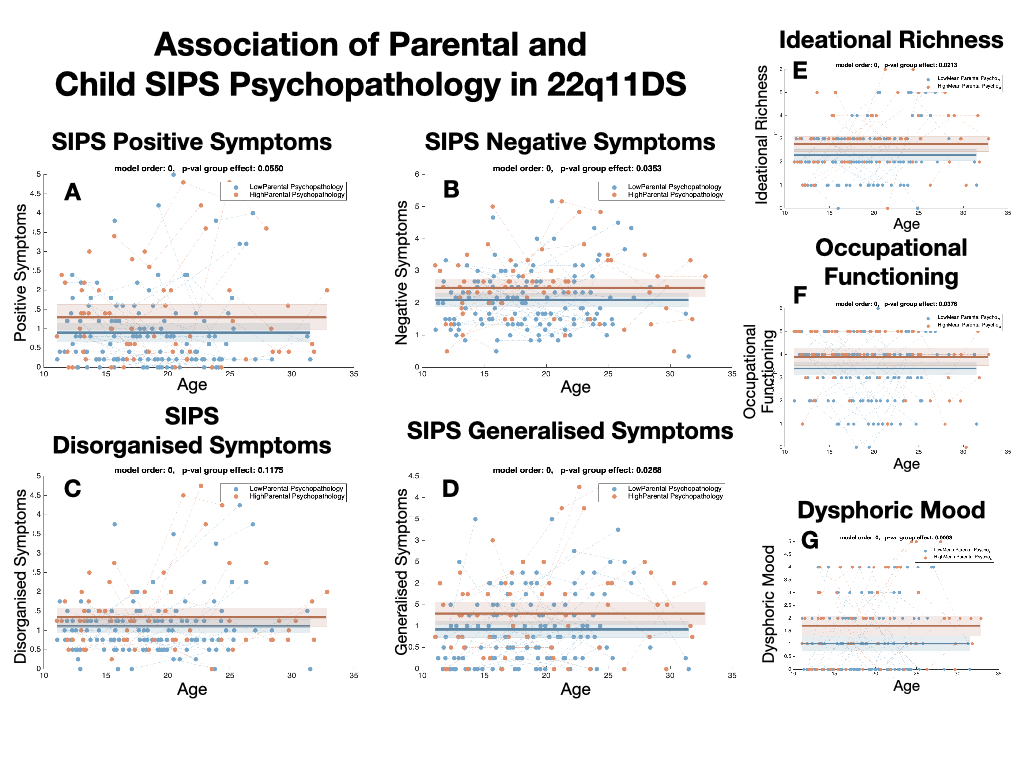
**

**Supplementary Figure 7:** Comparison of the trajectories of child SIPS psychopathology across sub-group of 22q11DS individuals, divided according to levels of parental psychopathology. **A:** SIPS positive symptoms**. B:** SIPS negative symptoms**. C:** SIPS disorganized symptoms**. D:** SIPS disorganized symptoms. **E:** SIPS ideational richness **F:** SIPS occupational functioning **G:** SIPS disphoric mood.
